# The role of cell cycle-associated protein 1 (CAPRIN1) in diseases

**DOI:** 10.7717/peerj.21772

**Published:** 2026-10-27

**Authors:** Chunmei Li, Peipei Tang, Yufeng Fu, Runyuan Ji

**Affiliations:** The Digestive and Reproductive System Cancers Precise Prevention Engineering Research Center of Jiangsu Province, Jiangsu College of Nursing, Huai’an, Jiangsu, China

**Keywords:** CAPRIN1, LLPS, SGs, Tumor, Viral infection, Neurological disease

## Abstract

Cell cycle associated protein 1 (CAPRIN1) is a widely expressed protein that is essential for many physiological and pathological processes. This review summarizes its structural features as well as regulation and functions and examines its role in tumors, viral infections, and neurological disorders. CAPRIN1 modulates cellular stress by regulating the dynamic assembly and disassembly of stress granules *via* liquid–liquid phase separation. The expression and function of CAPRIN1 are regulated at multiple regulatory layers, including transcriptional control, post-transcriptional regulation, RNA modification, and post-translational modifications. These findings discussed herein highlight the potential of CAPRIN1 as a diagnostic biomarker and therapeutic target, providing a theoretical basis for the treatment of tumors, viral infections, and neurological diseases.

## Introduction

The cell cycle-associated protein 1 (*CAPRIN1*) gene is located on the short arm of human chromosome 11 (11p13). It encodes a 709 amino-acid protein with a molecular weight of approximately 116 kDa (Grill et al., 2004). Although it is mainly localized in the cytoplasm, Okano et al. (2023) reported that CAPRIN1 is also present on the membranes of different cancer cells, but not on those of normal tissues. Considering its unique expression pattern, CAPRIN1 may be targeted by antibody-based anticancer drugs.

As an RNA-binding protein, CAPRIN1 can regulate various metabolic processes of RNA. For example, it regulates the reversible assembly of stress granules (SGs), which are dynamic cytoplasmic aggregates of RNA-binding proteins, translation-stalled mRNAs, translation initiation factors, and related proteins, that play a key role in cellular stress response (Anderson & Kedersha, 2009; Song et al., 2022). Additionally, CAPRIN1 controls the stability, translation, and transport of mRNAs encoding cell cycle-related proteins (Zhang & Peng, 2023). It also reportedly binds to *CCND2* and *c-MYC* mRNAs, thereby affecting the G1/S transition and regulating the cell cycle progression (Wang, David & Schrader, 2005). In embryonic stem cells (ESCs), CAPRIN1 eliminates abnormal transcripts during the early ESC differentiation by regulating RNA degradation, thereby altering cell differentiation and gene expression (Viegas et al., 2022). Besides regulating RNA metabolism, CAPRIN1 is involved in many physiological and pathological processes. It plays a dual role during viral infection—it suppresses viral replication by triggering the immune response, but some viruses can hijack it to inhibit the formation of SGs and thereby create a favorable intracellular environment for viral replication (Biering et al., 2017; Kurhade et al., 2023). In the nervous system, CAPRIN1, which colocalizes with RNA granules in neuronal dendrites, influences neuronal development and synaptic function by regulating mRNA transport and local translation (Zhang & Peng, 2023). Overall, CAPRIN1 functions as a crucial regulator in tumor development, viral infections, and neurodegenerative diseases.

Although previous studies have explored the multifunctional characteristics of CAPRIN1, a comprehensive review that systematically integrates its functions across diverse physiological and pathological contexts remains lacking. Unlike previous reviews that have primarily focused on specific biological aspects of CAPRIN1 or individual disease contexts, this review provides an integrated perspective encompassing molecular and structural biology, RNA regulation, liquid–liquid phase separation (LLPS), and stress granule (SG) biology, and further connects these mechanisms with the roles of CAPRIN1 in tumors, viral infections, and neurological diseases. In addition, we highlight the potential therapeutic implications and translational prospects of targeting CAPRIN1. We first summarize the molecular characteristics and core RNA-related functions of CAPRIN1, followed by a detailed discussion of its mechanisms of action in various tumours, viral infections, and neurological diseases. Finally, we highlight potential CAPRIN1-targeted therapeutic strategies and key scientific questions that remain to be addressed.

## Survey methodology

A literature search was performed for the keyword “Caprin1” in PubMed, Google Scholar, and Web of Science from inception to May 2026. Additional manual searches were conducted by reviewing the reference lists of the included articles and relevant reviews. As the work gradually progressed, the eligibility criteria were defined as original research and review articles reporting the role of CAPRIN1 in regulating the dynamic balance of SG formation and disassembly through liquid–liquid phase separation (LLPS) in the context of cancer, viral infection, and neurodegenerative diseases. Conference abstracts and articles not available in full text were excluded because of their lack of full data and detailed methodological information. All identified records were exported to EndNote 20 for removal of duplicates. Two reviewers (Chunmei Li, first author, and Peipei Tang, second author) independently screened all identified titles/abstracts and subsequently the full texts using predefined criteria. Disagreements were adjudicated by the corresponding author (Runyuan Ji). After removing the duplicates and irrelevant articles, 99 articles were finally selected for this review.

### Structure of CAPRIN1

The human CAPRIN family consists of two members, namely CAPRIN1 and CAPRIN2. The distinctive feature of this family is that all its members contain two highly conserved homologous regions (HR1 and HR2), an E-rich region, RGG (arginine-glycine-glycine) boxes, and RG-rich sequences characteristic of RNA-binding proteins. The C-terminus of CAPRIN2 additionally contains a C1q-related domain. dCaprin, a *Drosophila* protein, contains only an HR1 region, RGG boxes, and RG-rich sequences, lacking the HR2 domain and E-rich region (Grill et al., 2004; Shiina, Shinkura & Tokunaga, 2005; Solomon et al., 2007; Song et al., 2022; Zhu et al., 2020). Although CAPRIN1 and CAPRIN2 shares several conserved structural features, this review focuses on CAPRIN1, whose roles in RNA metabolism, stress granule (SG) biology, LLPS, cancer, viral infection and neurological disorders have been more extensively characterized. The findings discussed here therefore primarily apply to CAPRIN1 and should not be generalized to the CAPRIN family without direct evidence. The 132–251 amino acid residues of CAPRIN1 fold into an all-α-helical structure and form a homodimer *via* self-interaction. This region forms a binding pocket with a highly negatively charged surface V-shaped groove, providing the structural basis for the binding of CAPRIN1 to different proteins (Wu et al., 2016; Zhu et al., 2020). For example, the RasGAP SH3 domain-binding protein (G3BP) 1 interacting motif (GIM), located within the 352–380 amino acid residues of the HR2 domain, mediates the binding of CAPRIN1 to G3BP1. The heterodimeric interaction of CAPRIN1 with G3BP1 enhances the ability of G3BP1 to recruit mRNA, thereby promoting SG assembly. As a core scaffold protein for SG assembly, G3BP1 plays a key role in cellular stress responses (Lavrynenko et al., 2025; Solomon et al., 2007; Song et al., 2022). In addition, the 231–245 amino acid residues of CAPRIN1 constitute the FMRP binding domain (Wu et al., 2016). Dimerized CAPRIN1 interacts with the Fragile X mental retardation protein (FMRP) and G3BP1, forming a CAPRIN1-FMRP-G3BP1 regulatory network (Zhu et al., 2020). This is very important for neuronal mRNA transport and local translation and thereby influences synaptic plasticity and neuronal structure. These processes are finely regulated by FMRP, which is a widely expressed RNA-binding protein (Richter & Zhao, 2021). Moreover, CAPRIN1contains a prion-like domain, which comprises the 537–709 amino acid residues. This low-complexity domain is rich in amino acid residues similar to those found in the yeast prion domain. The residues G624–R626, G638–R640, and R660–Q666 of CAPRIN1 are crucial for LLPS through their involvement in weak polyvalent interactions. This mechanism constitutes the basis for its participation in the formation of biomolecular condensates (March, King & Shorter, 2016; Zhou, 2025). Figure 1 shows the structural schematic diagrams of the CAPRIN family.

**Figure 1:**
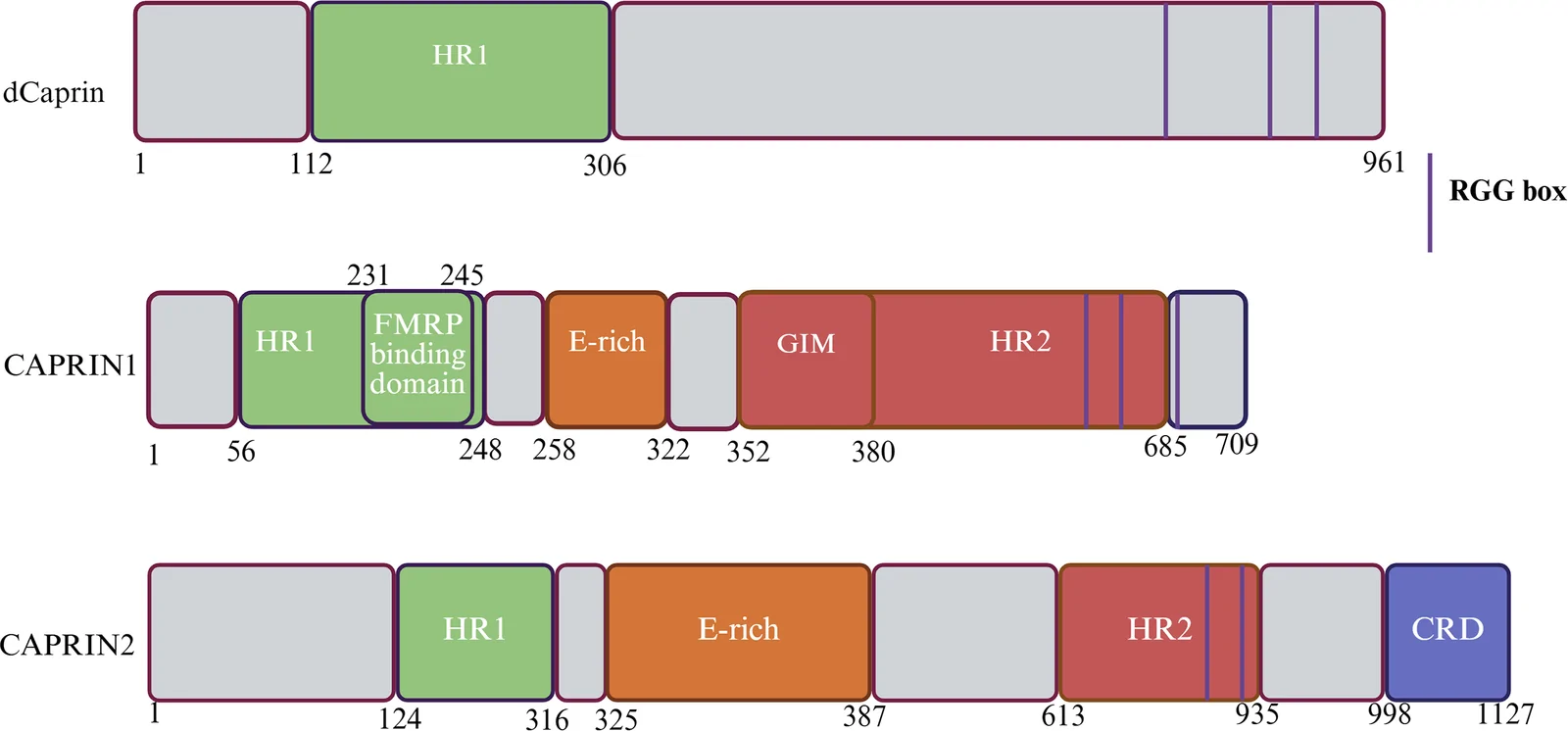
Schematic representation of the CAPRIN1 protein family. Schematic representation of dCaprin and human CAPRIN1 and CAPRIN2. HR1 and HR2: homologous regions 1 and 2; GIM: G3BP1-interacting motif. The purple lines indicate the locations of the RGG boxes. CAPRIN1, cell cycle associated protein 1. Created in Biorender.

### CAPRIN1-mediated phase separation

CAPRIN1 regulates the dynamic assembly of SGs through LLPS. In this process, biomolecules, such as proteins and nucleic acids, form membraneless organelles known as SGs through dynamic interactions under specific conditions (Zhang et al., 2020). SGs are cytoplasmic condensates formed by ATP-driven LLPS mechanisms in eukaryotic cells in response to stress factors, such as heat shock and oxidative stress (Jain et al., 2016; Protter & Parker, 2016). As a key regulator of SGs, CAPRIN1 coordinates their assembly and disassembly through interactions with G3BP1, mRNAs, and non-coding RNAs (ncRNAs) (Song et al., 2022). CAPRIN1 competes with ubiquitin-specific protease 10 (USP10) for binding to G3BP1. The G3BP1/CAPRIN1 complex promotes G3BP1-driven LLPS, thereby facilitating SG assembly. Conversely, USP10 induces SG disassembly by maintaining the soluble conformation of G3BP1. CAPRIN1 and USP10 competitively regulate LLPS mediated by G3BP1 through their GIM domains, which target the same hydrophobic pocket of G3BP1 (Kedersha et al., 2016). CAPRIN1 also exhibits an intrinsic “yin-yang” regulatory mechanism mediated by its N-terminal domain (NTD) and C-terminal domain (CTD). Specifically, the NTD of CAPRIN1 inhibits LLPS, whereas its CTD spontaneously induces LLPS in an RNA-dependent manner, thereby promoting the formation of SGs (Kim et al., 2021; Song et al., 2022). CAPRIN1 recruits protein arginine methyltransferase 1 (PRMT1) to SGs through its CTD. Under stress conditions, it enables PRMT1 to catalyze asymmetric dimethylarginine (ADMA) modification of G3BP1 at its RGG motifs, thereby inhibiting SG assembly. Conversely, Jumonji C domain-containing protein 6 functions as an arginine demethylase that reduces this ADMA modification of G3BP1 and consequently promotes SG formation. Mechanistically, CAPRIN1 acts as a molecular linker that couples PRMT1 with G3BP1, promoting the formation of a CAPRIN1-PRMT1-G3BP1 complex. In the absence of stress, CAPRIN1 adopts a molecular spacer role that restrains abnormal SG assembly (Qin et al., 2024; Tsai et al., 2016, 2017). Figure 2 summarizes the molecular mechanism underlying the regulation of SG dynamics by CAPRIN1 through LLPS.

**Figure 2:**
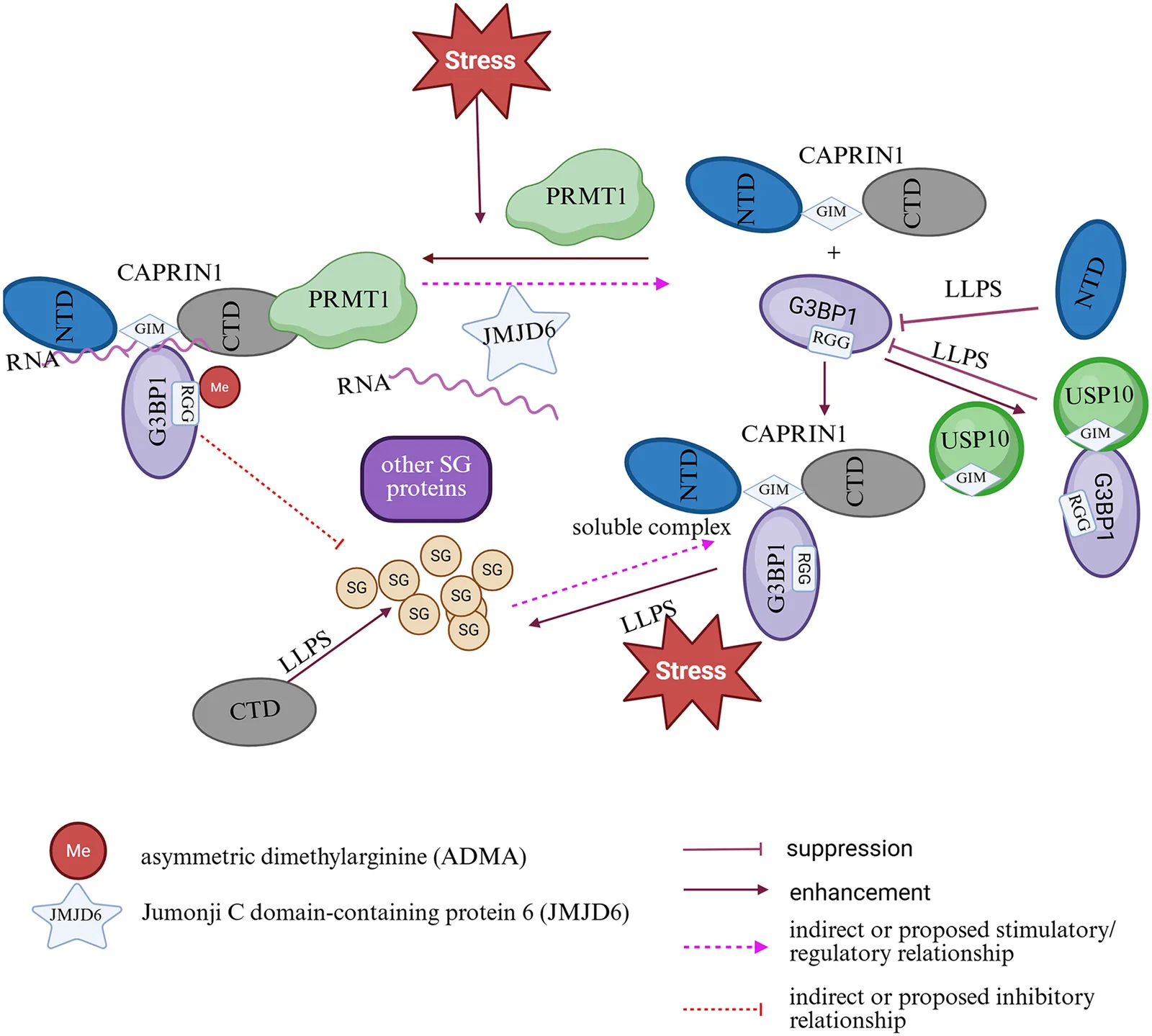
Proposed model of CAPRIN1-mediated regulation of SG dynamics through LLPS. This figure presents a proposed conceptual model integrating experimently supported interactions and mechanisms reported in different studies. CAPRIN1 promotes SG assembly through LLPS by binding to G3BP1, RNAs, and other SG-associated proteins. USP10 competes with CAPRIN1 for G3BP1 and drives SG disassembly by maintaining G3BP1 solubility. CAPRIN1 recruits PRMT1 into SGs and acts as a molecular linker to connect PRMT1 to G3BP1. Under stress conditions, PRMT1 catalyzes the ADMA of G3BP1 at its RGG motifs (Me). JMJD6 has been shown to reduce this ADMA modification of G3BP1, and consequently promotes SG formation. CAPRIN1 acts as a molecular spacer to prevent aberrant SG aggregation. The CTD of CAPRIN1 undergoes spontaneous LLPS, whereas the NTD of CAPRIN1 suppresses G3BP1-mediated LLPS. The overall integration of these mechanisms represents a proposed framework rather than an established unified pathway. CAPRIN1, cell cycle associated protein 1; LLPS, liquid–liquid phase separation; CTD, C-terminal domain; SG, stress granule; ADMA, asymmetric dimethylarginine; G3BP1, RasGAP SH3 domain-binding protein1; USP10, ubiquitin-specific protease 10; PRMT1, protein arginine methyltransferase 1; JMJD6, Jumonji C domain-containing protein 6. Created in Biorender.

## Regulation of CAPRIN1

### Transcriptional regulation

Studies on the transcriptional regulation of the *CAPRIN1* gene remain limited. Using a mouse model of ototoxic hearing damage, Towers et al. (2011) found that *Caprin1* is a direct target of the transcription factor *Pou4f3*, wherein Caprin1 is recruited to SGs of cochlear hair cells. *Pou4f3* negatively regulates the expression of *Caprin1* by directly binding to the functional promoter in the 5′-flanking region of *Caprin1*. Damage to hair cells reduces the Pou4f3 activity and relieves the transcriptional repression of *Caprin1*, leading to significant upregulation of its mRNA and protein levels. This process promotes SG formation and enhances hair cell survival by inhibiting the translation of pro-apoptotic gene mRNAs (Towers et al., 2011).

### Regulation of mRNA translation

#### Regulation of noncoding RNAs (ncRNAs)

Noncoding RNAs include long ncRNA (lncRNAs), microRNAs (miRNAs), and circular RNAs (circRNAs), all of which regulate the proliferation, metastasis, and survival of tumor cells by influencing the stability or translation efficiency of *CAPRIN1* mRNA (Towers et al., 2011).

##### LncRNAs

Several lncRNAs have been found to contribute to tumorigenesis and progression of tumors by binding to CAPRIN1 (Luo et al., 2021; Zhuo et al., 2022). In cisplatin-resistant A549 and H1299 cells, the lncRNA SNHG9 significantly promotes CAPRIN1 expression (Wang et al., 2020). Conversely, in nasopharyngeal carcinoma (NPC) cells, the lncRNA XIST is negatively correlated with the expression of CAPRIN1, which indicates that it may promote tumor progression by inhibiting CAPRIN1 (Lu et al., 2021). Moreover, in ovarian cancer (OC), the lncRNA SNHG8 positively regulates CAPRIN1 expression, and downregulation of the SNHG8/CAPRIN1 axis inhibits proliferation, migration, epithelial–mesenchymal transition (EMT), and stemness maintenance, while promoting apoptosis and β-catenin/Axin1 degradation. Mechanistic studies have shown that this axis functions through the regulation of the Wnt/β-catenin signaling pathway (Miao et al., 2020). Under glutamine-deprived conditions, CAPRIN1 binds to the 3′ untranslated region (UTR) of glutaminase 1 mRNA, whereas the lncRNA GIRGL promotes CAPRIN1 dimerization, thereby promoting LLPS and SG formation to support tumor cell survival in nutrient-deprived microenvironments (Wang et al., 2021).

##### CircRNAs

The covalently closed structure of circRNAs enables them to directly interact with RNA-binding proteins. For example, circ288 directly binds to Caprin1 and upregulates N-methyl-D-aspartate receptor subunit 3B mRNA expression, thereby alleviating neuronal damage caused by epilepsy, promoting hippocampal neurogenesis, and inhibiting abnormal neuronal migration in neuron-specific Caprin1 knockout mice (Guo et al., 2025). In gastric cancer (GC), circIPo7 acts as a competitive endogenous RNA to regulate CAPRIN1 expression, it directly binds to CAPRIN1 and disrupts its interaction with G3BP1, leading to the dissociation of CAPRIN1, epidermal growth factor receptor, and mammalian target of rapamycin (mTOR) mRNAs from the ribosomes, thereby inhibiting translation and inactivating the PI3K/AKT/mTOR signaling pathway (Liu et al., 2023). Furthermore, circVAMP3 enhances the interaction between CAPRIN1 and G3BP1, promoting SG assembly (Chen et al., 2022).

##### MiRNAs

Most miRNAs inhibit CAPRIN1 expression by binding to the 3′ UTR of its mRNA (Zhang et al., 2018). For example, miR-16 reduces CAPRIN1 protein levels or induces its mRNA degradation in HeLa and MCF7 cells (Kaddar et al., 2009). In colorectal cancer (CRC), miR-193a-3p enhances Argonaute2 (Ago2)-mediated *CAPRIN1* mRNA cleavage in a tryptophan-dependent manner, thereby inhibiting the expression of CAPRIN1 and of its downstream target genes *CCND2* and *c-MYC* (Teng et al., 2017; Xu et al., 2024). Similarly, miR-193a/b-3p acts as a therapeutic target by directly targeting *CAPRIN1* in liver fibrosis (Ju et al., 2019). In GC, miR-181a inhibits CAPRIN1 expression by binding to its 3′ UTR, thereby promoting tumor progression (Lu et al., 2018). In breast cancer, miR-223 inhibits CAPRIN1 expression by directly binding to the 3′ UTR of *CAPRIN1* mRNA, thereby inhibiting tumor cell proliferation and invasion (Gong et al., 2013). In renal cell carcinoma (RCC), miR-1 is significantly downregulated, which leads to weakened inhibition of CAPRIN1, cyclin-dependent kinase 4 (CDK4), and CDK6, thereby driving cell cycle progression. Conversely, overexpression of miR-1 inhibits the expression of these factors, induces G1/S phase arrest, and inhibits tumor cell proliferation, suggesting that the miR-1/CAPRIN1 axis represents a potential therapeutic target for RCC (Xiao et al., 2015).

#### Regulation of translational factors

In addition to ncRNAs, translational factors are also involved in the regulation of the expression of CAPRIN1. In lung adenocarcinoma (LADC), eukaryotic initiation factor 3 subunit m (EIF3M) positively regulates CAPRIN1 expression by binding to the 5′ UTR of its mRNA (Liu et al., 2021).

#### Regulation by RNA modifications

RNA modifications also play a significant role in the regulation of CAPRIN1 expression. In OC, N-acetyltransferase 10 stabilizes the *CAPRIN1* mRNA through N4-acetylcytidine (ac4C) modification, thereby enhancing its expression and promoting tumor cell invasion, migration, and maintenance of stemness (Song & Cheng, 2025).

### Post-translational modifications

CAPRIN1 undergoes various post-translational modifications (PTMs), including phosphorylation (Kim et al., 2019), O-GlcNAcylation (Drougat et al., 2012), and ubiquitination (Nakayama et al., 2017), which finely regulate its functional dynamics. The phosphorylation of tyrosine residues at the C-terminal region of CAPRIN1 modulates its phase-separation behavior, thereby influencing mRNA translation and stability (Kim et al., 2019). These modifications reduce its propensity for phase separation, consequently regulating its cellular functions (Drougat et al., 2012). The dynamic changes in O-GlcNAcylation levels may further regulate the assembly and disassembly of RNA granules containing CAPRIN1 in the cytoplasm (Kim et al., 2021). The speckle-type POZ protein (SPOP) promotes the ubiquitination of CAPRIN1, triggering its proteasome-dependent degradation. This process inhibits SG assembly, reduces stress-induced cell survival, and attenuates the resistance of prostate cancer (PC) cells to docetaxel (Shi et al., 2019). CAPRIN1 can also function as a component of the CAPRIN1/CUL1/FBXO42 E3 ubiquitin ligase complex, which promotes the ubiquitination and degradation of substrates such as small ubiquitin-like modifier 1 (Bellail et al., 2021).

## Role of CAPRIN1 in cancer

### Role of CAPRIN1 in digestive system cancers

CAPRIN1 is highly expressed in various gastrointestinal tumors and has been identified as a prognostic biomarker (Gao et al., 2024). Its oncogenic mechanisms are tumor-specific.

In esophageal carcinoma, CAPRIN1 facilitates malignant progression through metabolic reprogramming and resistance to ferroptosis (Zhang & Peng, 2023). Mechanistically, CAPRIN1 activates the glycolytic pathway by upregulating the expression of methyltransferase-like three and Wilms tumor 1-associated protein in ESCA cells thereby providing an energy advantage for cell proliferation (Gao et al., 2023). Additionally, CAPRIN1 knockdown significantly sensitizes ESCA cells to RAS-selective lethal three or erastin-induced ferroptosis, concomitant with pronounced elevation of reactive oxygen species (ROS) and malondialdehyde. Notably, CAPRIN1 expression correlates positively with ataxia telangiectasia mutated (ATM) transcript levels, and CAPRIN1 knockdown leads to ATM downregulation, though the functional contribution of this effect warrants further investigation (Gao et al., 2024). Beyond its cell-intrinsic functions, CAPRIN1 can directly bind to inhibitor of differentiation 4 (*ID4*) mRNA and promotes its degradation in ESCA cells. A coculture experment and a mouse xenograft model of ID4-transduced ESCA cells revealed that CAPRIN1-mediated *ID4* downregulation promotes cell proliferation and drives polarization of macrophages toward the M2 phenotype (Han et al., 2026). This finding extends the function of CAPRIN1 from direct regulation of the proliferation of tumor cells to the regulation of the tumor microenvironment. In hepatocellular carcinoma, CAPRIN1 knockdown downregulates the mRNA levels of *CCND1* and *CCND2*, which in turn impairs the proliferation and migration of HepG2 cells. This suggests that CAPRIN1 facilitates liver cancer progression by controlling the mRNA stability of cell cycle-related genes (Guo et al., 2020). Results from RNA immunoprecipitation (RIP) and RNA pull-down assays have demonstrated that RGG domain of CAPRIN1 engages *NCOA4* mRNA and promotes its recruitment into SGs, resulting in translational repression of *NCOA4* mRNA, which in turn suppresses ferritinophagy and lipid peroxidation. β-catenin can regulate *CAPRIN1* by directly binding to its promoter, thereby enhancing *CAPRIN1* transcription and amplifying SG formation. Furthermore, xenograft experiments provided *in vivo* evidence that CAPRIN1 depletion restores the expression of NCOA4 and renders resistant tumors responsive to sorafenib treatment (Wang et al., 2026).

In pancreatic ductal adenocarcinoma, CAPRIN1 significantly promotes tumor growth by activating autophagy. Mechanistically, results from co-immunoprecipitation (CO-IP) and mass spectrometry have demonstrated that CAPRIN1 interacts with Unc-51-like kinase 1 (ULK1) and serine/threonine kinase 38 to regulate ULK1 phosphorylation levels, thereby initiating the autophagy cascade in both Bxpc-3 and Panc-1 cells (Yang et al., 2023). This mechanism operates in parallel to the classical AMPK/mTORC1-ULK1/2 autophagy pathway, suggesting that CAPRIN1 may act as an alternative linker molecule between nutrient sensing and autophagy initiation independent of the canonical signaling axis (Chan, 2012; Yamamoto, Zhang & Mizushima, 2023). CAPRIN1 also acts as an oncogene in CRC. For example, tryptophan deprivation enhances the cleavage of *CAPRIN1* mRNA mediated by Ago2, thereby downregulating CAPRIN1 expression by targeting the nucleotides 41–49 in its 3′ UTR and consequently inhibiting its downstream targets *CCND2* and *c-MYC* using molecular and cellular experiments together with a mouse model of CRC liver metastasis (Xu et al., 2024).

In summary, CAPRIN1 exerts oncogenic effects in gastrointestinal tumors through multiple mechanisms, including metabolic reprogramming, resistance to ferroptosis, translational regulation, autophagy activation, and post-transcriptional regulatory networks. Although these mechanisms are tumor-specific, as an RNA-binding protein, CAPRIN1 exerts essential control over mRNA stability and translational efficiency of key genes.

### Role of CAPRIN1 in respiratory system cancers

CAPRIN1 also plays an oncogenic role in respiratory system cancers. The mechanism in laryngeal squamous cell carcinoma (LSCC), NPC, LADC, and non-small cell lung cancer (NSCLC) involve various aspects, such as glucose metabolism reprogramming, translational regulation, and chemoresistance.

In LSCC, the high expression of CAPRIN1 in tumor tissues is associated with poor prognosis. The results from RNA pull-down and RIP assays show that CAPRIN1 enhances the expression of Zic family member five by directly binding to the 3′ UTR of its mRNA, thereby increasing its stability and translation efficiency. This pathway promotes glycolytic metabolism (evidenced by elevated 2-deoxy-D-glucose and lactate levels) and drives the development of chemoresistance (Zhang et al., 2022). In NPC, CAPRIN1 promotes malignant progression through multiple mechanisms. Upregulation of the expression of CAPRIN1 increases the stability of *CCND2* mRNA to drive the cell cycle, induces EMT to enhance migratory ability, and inhibits cell apoptosis by downregulating cleaved PARP and cleaved caspase-3. Notably, downregulation of the expression of CAPRIN1 significantly inhibits these malignant phenotypes and enhances the sensitivity of NPC cells to rapamycin, cisplatin, and X-ray irradiation (Yang et al., 2022). In LADC, results from RIP, RNA pull-down, and dual-luciferase reporter assays have shown that overexpression of EIF3M upregulates CAPRIN1 expression at the post-transcriptional level by binding to the 5′ UTR of *CAPRIN1* mRNA. Ubiquitin C-terminal hydrolase L5 stabilizes the EIF3M protein through deubiquitination, thereby promoting the proliferation, invasiveness, and anti-apoptotic ability of LADC cells, and is associated with poor prognosis in TCGA datasets (Liu et al., 2021). Furthermore, CAPRIN1 is present in exosomes derived from patients with NSCLC, which indicates its potential as a non-invasive liquid biopsy biomarker for the diagnosis and monitoring of NSCLC (Durendez-Saez et al., 2022).

### Role of CAPRIN1 in reproductive system cancers

The mechanisms underlying the role of CAPRIN1 in reproductive system cancers are also multifactorial, including transcriptional activation, and signaling pathway integration.

As a transcriptional activator of phospholipid phosphatase 4 (PLPP4), CAPRIN1 is significantly upregulated in breast cancer tissues. PLPP4 overexpression reverses the anti-proliferative, anti-migratory, and pro-apoptotic effects and cell sensitization to doxorubicin caused by *CAPRIN1* silencing in HCC 1937 and MDA-MB-231 cells, which confirms that CAPRIN1 promotes breast cancer progression and chemoresistance through PLPP4 (Yuan & Yang, 2025). In triple-negative breast cancer (TNBC), the endogenous programmed death ligand 1 (PD-L1)/CAPRIN1 complex promotes cancer cell migration and metastasis. The mechanism involves PD-L1 internalization induced by inhibition of ceramide synthase 4/ceramide. The internalized PD-L1 binds to CAPRIN1 to form a complex that can stabilize the *SHH*, *TGFBR1*, and *Wnt* mRNAs, activating the Wnt/β-catenin signaling pathway and ultimately promoting the growth and metastasis of TNBC in clinical samples and databases (Wofford et al., 2024). This finding directly links the function of CAPRIN1 to the immune-related molecule PD-L1, expanding our understanding of its oncogenic mechanisms. In both TNBC cell-derived and patient-derived xenograft models, intercellular sclerostin acts as an intracellular oncogenic driver that interacts with CAPRIN1 to stabilize CDK and cyclin B1 mRNA (Sun et al., 2026). In cervical cancer (CC), CAPRIN1 upregulates the expression of HSP90α through the transcription factor YY1 in Hela/DDP and SiHa/DDP cells. The subsequent HSP90α–isocitrate dehydrogenase-1 (IDH1) interaction prevents the ubiquitination and degradation of IDH1, thereby stabilizing it. Elevated IDH1 levels promote NADPH biosynthesis and concurrently reduce ROS levels, which in turn suppress ferroptosis and fosters cisplatin resistance. Conversely, knockdown of *CAPRIN1* attenuates the YY1/HSP90α axis, accelerates IDH1 degradation, and ultimately activates ferroptosis and restores the cisplatin sensitivity of CC cells (Xie et al., 2026).

### Role of CAPRIN1 in urinary system cancers

The regulatory mechanisms of CAPRIN1 in urinary system cancers are context-dependent. Specifically, in PC, its expression has been reported to be regulated by the ubiquitin-proteasome pathway, whereas in renal cell carcinoma, it is primarily regulated by ncRNAs. In PC, the stability of CAPRIN1 protein is regulated by the ubiquitination regulatory network in a dual manner. For example, SPOP is a substrate adaptor for the CULLIN3/RING-box1 E3 ubiquitin ligase complex, which can recognize and bind to CAPRIN1, thereby promoting its ubiquitin-dependent degradation and inhibiting SG assembly. Importantly, mutations in the *SPOP* gene disrupt this degradation process, leading to CAPRIN1 accumulation, which in turn enhances chemoresistance to docetaxel and paclitaxel in a CAPRIN1-dependent manner. These findings are supported by evidence from cell lines, patient samples, and xenograft models in PC (Shi et al., 2019). In a distinct context, CAPRIN1 may also contribute to tumor-suppressive activity by serving as a component of the CAPRIN1/CUL1/FBXO42 E3 ubiquitin ligase complex, which promotes the ubiquitination and degradation of substrates such as small ubiquitin-like modifier 1 (SUMO1), thereby potentially suppressing tumorigenesis (Bellail et al., 2021; Zhang & Peng, 2023).

In summary, CAPRIN1 plays context-dependent roles in digestive, respiratory, reproductive, and urinary system tumors. As an RNA-binding protein, it regulates diverse tumor behaviors, including proliferation, apoptosis, invasion, metastasis, metabolic reprogramming, immune evasion, and treatment resistance of tumor cells through multiple oncogenic signaling pathways (Zhou et al., 2023). These different functions may depend on cellular context, interacting proteins, subcellular localization, and the regulatory mechanisms engaged by CAPRIN1. These findings highlight the value of CAPRIN1 as a prognostic or diagnostic biomarker for various cancers, while also indicating that its biological and clinical significance should be interpreted in a context-dependent manner. Table 1 provides a detailed summary of the function of CAPRIN1 in different cancers.

**Table 1:** The function of CAPRIN1 in different cancers.

Cancer type	Expression level	Mode of regulation	References
Digestive system cancers	UP	CAPRIN1 drives cell proliferation and glycolytic metabolism in ESCA by upregulating METTL3 and WTAP—core subunits of the N6-methyladenosine (m6A) methyltransferase complex.	Gao et al. (2023)
	UP	CAPRIN1 attenuates RAS-selective lethal 3-(RSL3) and erastin-induced ferroptosis in ESCA.	Gao et al. (2024)
	UP	CAPRIN1 binds to ID4 mRNA and downregulates its stability. ID4 inhibits ESCA proliferation and macrophage M2 polarization.	Han et al. (2026)
	UP	lncRNA ZFAS1 enhances invasive and proliferative capacities in GC by upregulating the expression of LIN28 and CAPRIN1.	Zhuo et al. (2022)
	DOWN	miR-181a promotes gastric cancer cell proliferation, invasion, and metastasis by directly suppressing CAPRIN1 expression in preclinical models of GC.	Lu et al. (2018)
	UP	circIP07 directly binds to CAPRIN1, disrupting the CAPRIN1–G3BP1 interaction and dissociating CAPRIN1 along with EGFR and mTOR mRNAs from ribosomes, thereby suppressing the PI3K/AKT/mTOR signaling axis in GC.	Liu et al. (2023)
	UP	miR-621 attenuates HCC progression by directly silencing CAPRIN1, which disrupts oncogenic signaling *via* downregulation of c-MYC and CCND2, which are key drivers of cell cycle progression.	Zhang et al. (2018)
	UP	CAPRIN1 promotes HCC progression by transcriptionally activating *CCND1* and *CCND2*, thereby accelerating cell cycle progression and invasiveness through cyclin-dependent signaling pathways.	Guo et al. (2020)
	UP	circVAMP3 suppresses HCC cell proliferation and metastasis by driving CAPRIN1 phase separation, which attenuates c-MYC translation and facilitates SG assembly.	Chen et al. (2022)
	UP	lnc-Ma301 suppresses HCC metastasis and epithelial–mesenchymal transition (EMT) by forming a functional complex with CAPRIN1 to inhibit the Akt/ERK1 signaling pathway.	Luo et al. (2021)
	UP	The RGG box of CAPRIN1 interacts with *NCOA4* mRNA and recruits it into SGs, leading to the repression of *NCOA4* translation and inhibition of sorafenib-induced ferroptosis in HCC.	Wang et al. (2026)
	UP	CAPRIN1 interacts with both ULK1 and STK38 in PDAC, driving autophagy activation and pro-tumorigenic phenotypes through ULK1 phosphorylation-dependent mechanisms, while enabling immune evasion.	Yang et al. (2023)
	UP	The Trp/Ago2/miR-193a-3p axis promotes RNA-induced silencing complex (RISC) activity in CRC by stabilizing the catalytic conformation of Ago2 *via* Trp-pocket engagement. This facilitates *CAPRIN1* mRNA degradation and coordinated downregulation of oncogenic effectors (COX2, CCND2, c-MYC, and β-catenin), impairing tumor growth and survival.	Xu et al. (2024)
	UP	miR-193a attenuates CRC growth by silencing CAPRIN1, which disrupts oncogenic signaling cascades and reduces *CCND2* and *c-MYC*-driven cell cycle advancement.	Teng et al. (2017)
Respiratory system cancers	UP	CAPRIN1 enhances glycolytic flux and chemotherapeutic resistance in LSCC through ZIC5-dependent regulatory networks.	Zhang et al. (2022)
	DOWN	lncRNA XIST promotes NPC progression by suppressing CAPRIN1 expression, which concurrently enhances tumor cell proliferation and metastatic dissemination.	Lu et al. (2021)
	UP	CAPRIN1 orchestrates NPC progression *via* dual oncogenic mechanisms: CCND2-driven cell cycle acceleration and EMT-mediated invasiveness; and apoptotic evasion through repression of PARP/caspase-3 cleavage. Genetic silencing of CAPRIN1 reverses chemoradiotherapy resistance, sensitizing tumors to DNA-damaging agents (cisplatin and X-rays) and mTOR inhibition (rapamycin).	Yang et al. (2022)
	UP	Targeting the UCHL5/EIF3M/CAPRIN1 signaling node attenuates malignant progression in LADC.	Liu et al. (2021)
	UP	lncRNA SNHG9 amplifies CAPRIN1-driven pro-survival signaling in NSCLC, which attenuates cisplatin-induced DNA damage response and apoptosis, ultimately fostering therapeutic resistance.	Wang et al. (2020)
Reproductive system cancers	UP	CAPRIN1-driven transcriptional upregulation of *PLPP4* impairs DOX efficacy in BCA by enhancing drug efflux and survival signaling, while accelerating proliferation, invasion, and apoptotic resistance.	Yuan & Yang (2025)
	UP	miR-223 attenuates BCA malignancy through direct targeting of CAPRIN1, which disrupts downstream proliferative and invasive pathways.	Gong et al. (2013)
	UP	The PD-L1/CAPRIN1 complex orchestrates a dual pro-tumorigenic program in TNBC: stabilization of *SHH/TGFBR1/WNT* transcripts amplifies β-catenin driven proliferation and metastasis; recruitment of immunosuppressive FOXP3(+) Tregs establishes an immune-excluded tumor microenvironment, blunting anti-PD-1/PD-L1 efficacy.	Wofford et al. (2024)
	UP	Intercellular sclerostin promotes TNBC progression and metastasis. It interacts with CAPRIN1 to stabilize *CDK1* and *Cyclin B1* mRNAs.	Sun et al. (2026)
	UP	The SNHG8/CAPRIN1 axis drives OC progression by coordinating pro-metastatic (EMT) and pro-survival (anti-apoptotic) programs. Surprisingly, CAPRIN1 depletion triggers a Wnt/β-catenin hyperactivation loop *via CTNNB1*/*AXIN1* mRNA destabilization.	Miao et al. (2020)
	UP	NAT10 orchestrates aggressive behaviors in OC through transcriptional activation of CAPRIN1, which amplifies EMT-driven metastasis and self-renewal pathways (*e.g*., Wnt/β-catenin).	Song & Cheng (2025)
	UP	CAPRIN1 knockdown promotes cisplatin sensitivity in cervical cancer (CC) by inhibiting the YY1/HSP90α axis, accelerating IDH1 degradation and activates ferroptosis.	Xie et al. (2026)
Urinary system cancers	UP	Wild-type SPOP (WT-SPOP) mediates the ubiquitin-dependent degradation of CAPRIN1 and suppressed stress granule (SG) assembly in PC.	Shi et al. (2019)
	UP	miR-1 suppressed cell proliferation and metastatic potential in RCC by directly targeting *CDK4*, *CDK6*, *CAPRIN1*, and *Slug*, which are key regulators of cell cycle progression and EMT.	Xiao et al. (2015)

### Role of CAPRIN1 in viral infections

CAPRIN1 plays a dual role in viral infection. Although it exerts antiviral effects by activating autophagy, interferon (IFN) signaling pathway, and disrupting SG assembly, it can be hijacked by certain viruses to promote their genome replication and inhibit SG formation (Biering et al., 2017; Hou et al., 2017; Kurhade et al., 2023; Reineke et al., 2015).

### Antiviral mechanisms

The cellular stress response constitutes the first line of defense for mammalian cells against viral infection (White & Lloyd, 2012). As a cellular stressor, viral infection can activate the host’s innate immune response. CAPRIN1 participates in this defense process through autophagy pathways and SG dynamics. Using cell lines and primary murine bone marrow-derived macrophages, Kurhade et al. (2023) found that CAPRIN1 not only interacts with autophagy-related 16-like protein 1 (ATG16L1), but also acts as a key regulator of murine norovirus (MNV) replication in an IFN gamma (IFNG)-dependent manner. MNV protects its RNA from the host innate immune defense by forming replication complexes (RCs). IFN-γ disrupts the RCs of MNV through autophagy proteins and inducible GTPases, with GBP1 being the only human GTPase that can target viral RCs. The covalent conjugation of ubiquitin-like microtubule-associated protein 1 light chain 3 (LC3) to phosphatidylethanolamine is essential for autophagy initiation, which is catalyzed by the ATG12-ATG5-ATG16L1 complex. LC3 is also required for the recruitment of IFN-γ-induced GTPases to RCs. Cell-based studies, together with *in vivo* mouse studies of the broader MNV replication-complex targeting mechanism have been showed that CAPRIN1 promotes the recruitment of lipidated LC3 to the RCs of MNV by interacting with G3BP1 and ATG16L1, thereby rendering these RCs susceptible to IFN-γ-induced GTPases (Biering et al., 2017; Kurhade et al., 2023). Furthermore, cell-based and molecular biochemical studies demonstrated that CAPRIN1 forms a complex with G3BP1 and protein kinase R (PKR), activating PKR and driving the phosphorylation of eukaryotic translation initiation factor 2α (eIF2α), thereby inhibiting translation and enhancing the production of type I IFN, which in turn activates the innate immunity response to combat viral infection (Yoo, 2024). In this process, G3BP1 is responsible for recruiting PKR to SGs, whereas CAPRIN1 directly interacts with PKR to regulate its activation state within SGs. Concurrently, CAPRIN1 plays a key role in releasing activated PKR into the cytoplasm to recognize substrates (Reineke et al., 2015). Additionally, evidence from HBV-infected cell models, primary human hepatocytes, humanized liver mice, and liver biopsy sample from patients identified CAPRIN1 as a major regulator governing the dynamics of the IFN response in the context of innate immunity and IFN-α adjuvant therapy against hepatitis B virus (HBV) infection/replication. Mechanistically, CAPRIN1 interacts with core SG proteins to drive SG formation in response to viral stress and facilitates the condensation of SGs surrounding the rG4 structure in the 5′ UTR of STAT1 mRNA, thereby suppressing STAT1 protein synthesis independently of its mRNA levels (Peng et al., 2026).

### Viral promotion mechanisms

In certain viral infection processes, CAPRIN1 is hijacked by the virus to promote viral replication. For example, using ZIKV-infected A549 cells and ZIKV protein-expressing HEK293T cells, Zika virus (ZIKV) infection induces cellular stress and activates PKR and the unfolded protein response (UPR), which in turn leads to eIF2α phosphorylation mediated by PKR and translation arrest. However, ZIKV capsid proteins can inhibit the formation of SGs by hijacking their core components, such as CAPRIN1 and G3BP1, while promoting viral replication. CAPRIN1 or G3BP1 knockdown significantly inhibits ZIKV replication (Hou et al., 2017). Similarly, cell-based infected and protein-interaction studies showed that during Japanese encephalitis virus (JEV) infection, the viral capsid protein interacts with CAPRIN1 to inhibit SG assembly, thereby causing translation arrest and enhanced viral replication (Katoh et al., 2013). CAPRIN1 and G3BP1 are essential factors for the intermediate transcription of vaccinia virus (VV) *in vitro* (Katsafanas & Moss, 2004). During wild-type VV infection, cell-based imaging studies showed that although CAPRIN1 and G3BP1 accumulate around the viral replication factories, they do not induce the formation of typical SGs (Katsafanas & Moss, 2007; Walsh et al., 2008). Notably, the E3L protein encoded by VV is a dsRNA-binding protein that can block the host antiviral response by sequestering dsRNA. When infected with mutant viruses lacking E3L, cellular infection studies showed that host cells activate PKR, phosphorylate eIF2α, and terminate translation initiation, thereby promoting the assembly of antiviral particles (AVGs) around the replication factories. AVGs contain the SG protein CAPRIN1, which can hinder viral replication at the transcriptional intermediate stage. Concurrently, AVGs recruit the antiviral protein TIA1 to directly inhibit viral gene expression (Chang, Watson & Jacobs, 1992; Langland & Jacobs, 2002; Simpson-Holley et al., 2011).

In summary, the dual role of CAPRIN1 during viral infection may be influenced by factors, such as virus type, cell type, and effector proteins encoded by the virus. For example, the capsid proteins of ZIKV and JEV can directly bind to CAPRIN1 to inhibit SG assembly, whereas VV utilizes the accumulation of CAPRIN1 in replication factories to promote its own transcription. The distinct outcomes are likely determined by the specific viral proteins and their interactions with CAPRIN1. Furthermore, the function of CAPRIN1 may be influenced by cell-specific co-regulators, such as G3BP1 and PKR, as well as by cellular stress conditions.

### Role of CAPRIN1 in neurological disorders

CAPRIN1 also plays a dual role in the nervous system. As a component of dendritic RNA granules, it is involved in synaptic plasticity and formation of long-term memory under normal physiological conditions. However, under pathological conditions, its dysfunction is closely associated with various neurodegenerative diseases and neurodevelopmental disorders (Zhang & Peng, 2023). In neurons, local translation within dendrites constitutes the molecular basis for synaptic plasticity and long-term memory formation, a process that is mediated by RNA granules within dendritic compartments (Shiina, Shinkura & Tokunaga, 2005). Results from mouse hippocampal neurons and animal behavioral models have been showed that CAPRIN1, also known as RNG105 and a component of RNA granules, plays a key role in the dendrites of hippocampal neurons. Its deficiency impairs the synaptic strength and structural plasticity of mouse hippocampal neurons, leading to severe impairments in long-term memory formation during spatial and contextual learning tasks (Nakayama et al., 2017).

CAPRIN1 is closely associated with cytoplasmic inclusions in various neurodegenerative diseases. In the cytoplasmic aggregates of motor neurons in the spinal cord and motor cortex of patients with amyotrophic lateral sclerosis (ALS), immunohistochemical and colocalization analyses showed that CAPRIN1 colocalizes with small nucleolar ribonucleoprotein 200. This interaction has also been verified in arsenite-induced SGs in HeLa and SH-SY5Y cells (Vu et al., 2021). The 607–707 amino acid-residue region within the CTD of CAPRIN1 acts as a scaffold for salt-dependent phase separation, as demonstrated by *in vitro* biochemical and phase-separation assars, creating a protein-rich solvent environment that destabilizes immature superoxide dismutase 1 and promotes its conversion to an aggregation-prone unfolded state, which is associated with familial ALS (Ahmed et al., 2024). Abnormal cytoplasmic aggregation of fused in sarcoma (FUS) and TAR DNA-binding protein 43 can alter the dynamic distribution of CAPRIN1, leading to its dissociation from RNA granules. Studies using neuronal cell models showed that this displacement disrupts mRNA stability and local translation around the granules, ultimately resulting in the loss of dendritic synapses (Horio et al., 2023). Furthermore, in Huntington’s disease, studies in striatal neuronal models showed that endoplasmic reticulum stress triggers the interaction of mutant huntingtin protein with CAPRIN1 and G3BP1 in striatal neurons, redistributing to RNA granules, thereby regulating the processing and translation of mRNAs encoding key proteins for neuronal and synaptic plasticity (Ratovitski et al., 2012).

Mutations in the *CAPRIN1* gene and alterations in its expression levels are also closely associated with neurodevelopmental disorders in addition to neurodegenerative diseases. The imbalance of SG dynamics is considered as one of the underlying pathogenic mechanisms. Using cellular and molecular interaction assays, missense mutations in the GIM domain of CAPRIN1 disrupt the interaction between CAPRIN1 and G3BP1, thereby inhibiting SG formation (Jia et al., 2022; Sheehan, Hampton & Madden, 2022). Additionally, genetic and clinical analyses of individuals with CAPRIN1 haploinsufficiency showed that CAPRIN1 haploinsufficiency leads to an autosomal dominant neurodevelopmental disorder, characterized by language impairments, hyperactivity, intellectual or learning disabilities, autism spectrum disorders, and associated respiratory or auditory complications (Pavinato et al., 2023). In cortical neurons derived from allogeneic induced pluripotent stem cells, cellular and functional studies showed that the CAPRIN1 P512L (Pro512Leu) mutation promotes protein misfolding and aggregation, leading to reduced neuronal activity and changes in SG dynamics. The overexpression of this mutant induces the accumulation of insoluble ubiquitinated aggregates that coaggregate with proteins implicated in neurodegenerative disorders, suggesting that the gain-of-function mutations of CAPRIN1 may give rise to a new form of early-onset neurodegenerative disease, clinically presenting with progressive ataxia and dementia (Bove et al., 2025; Delle Vedove et al., 2022).

In summary, the function of CAPRIN1 in the nervous system stems from its core ability as an RNA-binding protein, which regulates local mRNA translation. Under physiological conditions, it ensures synaptic plasticity by maintaining the integrity of dendritic RNA granules. In neurodegenerative diseases, the imbalance of protein dynamics leads to abnormal aggregation of SGs and loss of synapses. In neurodevelopmental disorders, gene mutations or changes in expression levels disrupt the assembly of SGs or induce protein misfolding, thereby leading to abnormal neuronal development. These findings collectively establish the critical role of the protein in maintaining the homeostasis of the nervous system and provide a theoretical basis for its use as both a diagnostic biomarker and therapeutic target in neurological diseases.

### “Yin-yang” regulation of SG dynamics by CAPRIN1

CAPRIN1 is a stress-responsive RNA-binding protein that governs mRNA translation and SG dynamics *via* LLPS. Under acute stress, CAPRIN1-mediated SG assembly promotes cell survival by modulating the translation and stability of selective mRNAs, thereby facilitating metabolic adaptation (Song et al., 2022; Zhang & Peng, 2023).

The C-terminal low-complexity region of CAPRIN1 acts as a phase-separation scaffold; its positively charged fragment undergoes salt-dependent phase separation, creating a highly concentrated proteinaceous condensate. The condensates can enrich specific subsets of “client” molecules (such as specific mRNAs and proteins) while excluding others. Alternatively, they can shield inter-protomer interactions of client proteins, delaying or completely suppressing their aggregation. Through selective regulation of their internal composition, condensates modulate the conformational states of resident proteins (Ahmed et al., 2024; Toyama et al., 2022). These findings support a proposed “yin-yang” model in which the regulation of CAPRIN1-mediated phase separation reflects a balance between electrostatic repulsion and interchain attractive interactions, modulated by factors, such as salt concentration, pH, ATP, nucleotides, and PTMs (Ahmed et al., 2025; Kim et al., 2021; Lin et al., 2025; Schulte et al., 2023; Toyama et al., 2023; Tsanai & Head-Gordon, 2025). This model is based on specific experimental observations of CAPRIN1-mediated phase separation and should be regarded as a conceptual framework rather than a universal mechanism governing all CAPRIN1 functions.

When the core mechanism encounters distinct disease microenvironments, the delicate balance may be disrupted in a context-dependent manner, leading to aberrant SG dynamics that ultimately drive disease. In the tumor microenvironment, under stress conditions, CAPRIN1-mediated SGs are pathologically sustained as a persistent cytoprotective barrier that stabilizes oncogenic transcripts, thereby promoting chemoresistance and immune evasion (Gao et al., 2024; Wofford et al., 2024; Xie et al., 2026). During viral infections, the role of CAPRIN1 exemplifies the host–virus arms race. For instance, CAPRIN1-assembled SGs enrich key components of antiviral signaling pathways, including proteins and mRNA involved in IFN production and autophagy initiation, thereby establishing an antiviral defense in the host (Biering et al., 2017; Kurhade et al., 2023; Peng et al., 2026; Reineke et al., 2015). Conversely, the mRNA or capsid proteins of certain viruses may directly bind to CAPRIN1 to inhibit SG assembly or facilitate RC formation, thereby generating a cellular environment favorable for viral replication (Bidet, Dadlani & Garcia-Blanco, 2014; Hou et al., 2017; Katoh et al., 2013; Kurhade et al., 2023; Peng et al., 2026). In neurodegenerative diseases, aberrant transformation of SGs from dynamic liquid droplets into solid-like condensates is a hallmark pathological feature, which then drives the proteotoxic aggregation that underpins synaptic failure (Ahmed et al., 2024; Toyama et al., 2022).

Importantly, not all CAPRIN1 functions are necessarily dependent on SG formation or LLPS. CAPRIN1 also regulates RNA stability, mRNA translation, RNA transport, and protein-protein interactions through mechanisms that may occur independently of mature SG assembly or large-scale phase separation. Thus, SG/LLPS-dependent mechanisms represent an important aspect of CAPRIN1 biology, but they should be distinguished from its SG/LLPS-independent functions.

Therefore, the "yin-yang" model should be viewed as a context-dependent conceptual framework for understanding CAPRIN1-mediated SG dynamics rather than a comprehensive mechanism for all CAPRIN1-dependent processes. Elucidating this context-specific regulatory network is critical for developing targeted therapeutic strategies that modulate SG dynamics without perturbing physiological stress adaptation.

## Conclusions

CAPRIN1 functions as a dynamic regulator of mRNA translation, stability, and trafficking, thereby contributing to both physiological processes—such as cell cycle regulation and SG assembly and disassembly—and pathological conditions, including neurodegenerative diseases, chemoresistance, viral infection, and tumor progression.

CAPRIN1 has been identified as a central modulator of drug resistance, which is a major obstacle in cancer treatment (Yang et al., 2019). Accumulating evidence has implicated CAPRIN1 in the malignant progression of various cancer types. CAPRIN1 present in plasma exosomes may serve as a potential liquid biopsy biomarker for early cancer diagnosis and drug resistance monitoring (Durendez-Saez et al., 2022). Importantly, whereas CAPRIN1 is normally confined to the cytoplasm in non-malignant cells, it has been reported to localize to the plasma membrane in a range of tumor cells and solid tumors. providing a distinct extracellular epitope that can be exploited for antibody-based targeting (Okano et al., 2023). Accordingly, CAPRIN1-targeting strategies include preclinical approaches targeting intracellular CAPRIN1 and SG-related mechanisms, as well as antibody-based targeting of surface-exposed CAPRIN1. A humanized anti-CAPRIN1 antibody (TRK-950) and SG-inhibiting peptides (TAT-SIP-C1/2 and SIP-U1-Antp) have provided preclinical proof-of-concept for targeting CAPRIN1 to reverse chemoresistance (Li et al., 2024; Okano et al., 2023). In contrast to these predominantly preclinical approaches, TRK-950 has entered clinical development as a first-in-class antibody directed against surface-exposed CAPRIN1 (Majima et al., 2025; Nishio et al., 2025).

Mechanistically, TRK-950 is designed to bind surface-exposed CAPRIN1 and engage immune effector functions, including antibody-dependent cellular cytotoxicity and antibody-dependent cellular phagocytosis, rather than directly inhibiting the intracellular CAPRIN1 pool or SG formation. Importantly, TRK-950 has also entered early clinical evaluation. In a first-in-human phase I study, TRK-950 showed good tolerability, no dose-limiting toxicities, and dose-dependent exposure, with the maximum tolerated dose not reached. Preliminary antitumor observations included stable disease and tumor cavitation in one patient with cholangiocarcinoma (Cassier et al., 2025). Subsequent clinical evaluation has also explored combination therapy, including nivolumab. However, these early clinical observations remain insufficient to establish definitive antitumor efficacy or the optimal combination strategy (Koyama et al., 2026).

Several uncertainties regarding CAPRIN1 expression and heterogeneity remain. Surface CAPRIN1 expression may vary among tumors and may not directly reflect total or intracellular CAPRIN1 abundance. Therefore, the relationships among surface CAPRIN1 expression, target engagement, and clinical response require further investigation. Moreover, the clinical development of TRK-950 should be distinguished from preclinical strategies targeting intracellular CAPRIN1, SG assembly, or LLPS, and its clinical activity should not be interpreted as direct evidence that intracellular CAPRIN1-mediated chemoresistance or SG dysregulation can be reversed. In striking contrast, in the fields of viral infection and neurodegenerative diseases, despite extensive experimental evidence elucidating the central role of CAPRIN1 in pathological SG conversion and the host–virus arms race, targeted intervention strategies have largely remained confined to the preclinical stage, and clinical translational breakthrough have yet to be achieved.

CAPRIN1 exhibits a context-dependent “yin-yang” regulatory role in SG dynamics, which should be regarded as a conceptual model rather than a universal mechanism, presenting both opportunities and challenges for therapeutic intervention. Systemic inhibition of intracellular CAPRIN1 may potentially affect physiological stress adaptation and antiviral responses, whereas antibody-based targeting of surface CAPRIN1 represents a distinct therapeutic strategy. Therefore, future studies should focus on clarifying the determinants and heterogeneity of CAPRIN1 surface expression, developing selective CAPRIN1 modulators and delivery strategies, and identifying companion biomarkers for patient selection and treatment monitoring. However, several additional challenges remain. First, because CAPRIN1 is equally indispensable for neuronal stress adaptation and antiviral innate immunity, systemic inhibition may exacerbate neurodegenerative pathology or compromise host antiviral defense (Pavinato et al., 2023). Second, the functional outcome of SGs—whether pro-survival or cytotoxic—is critically dictated by the disease microenvironment. While inhibiting SG assembly may reverse chemotherapy resistance, broad disruption of SG formation could also interfere with stress-adaptive mechanisms in normal cells. Therefore, achieving therapeutic efficacy requires precise regulation of the equilibrium between SG assembly and disassembly—a level of fine-tuning that current small molecules and antibodies have yet to accomplish. Third, the lack of selective delivery platforms renders it difficult to precisely confine CAPRIN1-targeted intervention to the tumor microenvironment, sites of viral infection, or neurodegenerative lesion areas while adequately sparing normal cells. Given these challenges, the therapeutic goal is not simply to suppress CAPRIN1-mediated SG assembly, but rather to restore the dynamic equilibrium of CAPRIN1-mediated SG assembly and disassembly, thereby preventing their irreversible transition into pathological aggregates.

Future research should prioritize the following directions: (i) obtaining high-resolution structures of full-length CAPRIN1 and its disease-associated aggregates using cryo-electron microscopy to elucidate the spatial conformation and physicochemical properties of its intrinsically disordered regions and to identify viral protein-binding pockets; (ii) developing selective modulators capable of distinguishing pathological SGs from normal physiological condensates; (iii) designing selective delivery platforms—such as antibody–drug conjugates, nanoparticle formulations, or protease-activatable prodrugs—to precisely restrict CAPRIN1-targeted intervention to the tumor microenvironment, sites of viral infection, and neurotoxic protein aggregates in neurodegenerative diseases, thereby avoiding systemic disruption of the normal physiological functions of CAPRIN1; (iv) developing and validating companion diagnostic methods based on exosomes or circulating tumor cells to enable biomarker-guided patient stratification across different disease contexts.

In summary, CAPRIN1 represents a promising but incompletely validated therapeutic target, with surface CAPRIN1 antibody targeting having entered early clinical development, while most intracellular CAPRIN1/SG/LLPS-targeting strategies remain preclinical. Further studies are needed to determine whether selective modulation of different CAPRIN1 pools can restore spatiotemporal SG homeostasis and achieve effective and safe therapeutic outcomes.

## Supplemental Information

10.7717/peerj.21772/supp-1 Supplemental Information 1 PRISMA 2020 flow diagram detailing the identification, screening, and inclusion processes. Initial searches in PubMed (*n* = 204), Web of Science (*n* = 220), and Google Scholar (*n* = 63) yielded 487 records, with two additional records identified through citation searching. After removing 255 duplicates using Endnote 20, 232 records were screened. Exclusions were based on irrelevance to the review (*n* = 148), conference abstracts (*n* = 32), corrections (*n* = 7), and retracted articles (*n* = 2). Following full-text assessment, 100 articles (92 research and eight review articles) were included in the final systematic review.

## Additional Information and Declarations

### Data Availability

The following information was supplied regarding data availability:

This is a Literature Review.
